# The effects of exergames for cognitive function in older adults with mild cognitive impairment: a systematic review and metaanalysis

**DOI:** 10.3389/fneur.2024.1424390

**Published:** 2024-07-16

**Authors:** Xiaowan Cai, Liya Xu, Hanyue Zhang, Tingting Sun, Jingjing Yu, Xiao Jia, Xiao Hou, Ruizhe Sun, Jian Pang

**Affiliations:** ^1^Faculty of Sports and Human Sciences, Beijing Sports University, Beijing, China; ^2^Key Laboratory of Sports and Physical Health, Ministry of Education, Beijing, China; ^3^College of Education, Zhejiang University, Hangzhou, Zhejiang, China; ^4^School of Physical Education, Northeast Normal University, Jilin, China; ^5^China Institute of Sports and Health, Beijing Sports University, Beijing, China; ^6^Tibet Institute of Sport Science, Tibet, China; ^7^Shuren Academy, The Affiliated High School of Peking University, Beijing, China

**Keywords:** cognitive ability, mild cognitive impairment, elder, exergames, metaanalysis, RCT

## Abstract

**Purpose:**

Exergames are an innovative method that can promote neuroplasticity and improve the cognitive abilities of the elderly. This study aimed to compare the effects of single-task and multi-task exergames on the cognitive ability of the elderly with mild cognitive impairment (MCI).

**Methods:**

Computerized literature search was performed using PubMed, Web of Science, EBSCO, Elsevier, ProQuest, China National Knowledge Infrastructure (CNKI), Wanfang and VIP database to identify relevant articles from the establishment of the database from inception to April 1, 2024. The inclusion criteria were: (i) participants aged 60 or older diagnosed with mild cognitive impairment, regardless of gender; (ii) use of randomized controlled trials (RCTs); (iii) interventions involving exergames with physical activity or as the primary variable; and (iv) outcome measures using standardized neuropsychological instruments to assess cognitive function, including statistical data on sample size, mean, and standard deviation. Finally, the included study comprised a total of 526 participants. Mean difference (MD) and 95% confidence interval (CI) were used to synthesize the effect size in the data.

**Results:**

11 studies were included. Due to the differences in the intervention methods, subgroup analysis was performed on the included research. Compared with the control group assessed by the Montreal Cognitive Assessment Scale, the single-task intervention improved the cognitive ability of the elderly with MCI (MD 3.40, 95% CI 2.43–4.37), the Mini-Mental State Examination Scale (MD 2.38, 95% CI −2.03 to 2.72), the Trail Making Test (MD −3.89, 95% CI −6.45 to −1.33), and the Digit Span Forward test (MD 1.16, 95% CI 0.73–1.60).

**Conclusion:**

This meta-analysis supports that exergames could be an effective cognitive rehabilitation method for MCI patients. Our study recommends that patients implement a customized exergames program and adhere to it for a long time. It is necessary to pay attention to the exercise guidelines and provide evidence from clinicians.

**Strengths and limitations of this study:**

(1) This meta-analysis supports that exergames could be an effective cognitive rehabilitation method for MCI patients. Our study recommends that patients implement a customized exergames program and adhere to it for a long time. It is necessary to pay attention to the exercise guidelines and provide evidence from clinicians. (2) This research provides preliminary evidence for the clinical utility of VR tasks developed for mild cognitive impairment. (3) In this paper, only relevant studies in Chinese and English were searched, and no studies in other languages were searched.

## 1 Introduction

As the global population aging has become an important public health problem, more and more families and individuals are affected by cognitive impairment. Mild cognitive impairment (MCI) was proposed by Peterson and his colleagues in 1997, which refers to the decline of individual cognitive ability beyond expectation based on age and education level. Still, the impact on daily life could be more obvious (1). Dementia has a more serious decline in cognitive ability and a wide range of symptoms, which will significantly impact the independence and quality of life of individuals and is described as a worldwide epidemic (2). Worldwide, the prevalence of mild cognitive impairment (MCI) in community-dwelling adults aged 50 and older is about 15.56% (3). According to the American Academy of Neurology, MCI affects 8% of those aged 65–69, 15% of those aged 75–79, 25% of those aged 80–84, and 37% of those aged 85 and older (4). People with MCI may experience difficulties with memory, such as losing things often, having difficulty recalling names or words, missing appointments, and having a harder time finding familiar places and keeping track of important dates (5). Follow-up studies have shown that MCI patients have a 5–10% conversion rate of dementia each year, which is much higher than the incidence of 1–2% per year in the general population (6). Age is the biggest risk factor for MCI. According to the different folks involved in the study, the incidence of MCI in people over 65 years old is 3–22% (7–9), mainly manifested as memory, attention loss, and cognitive impairment (10). The brain began to change a few years before obvious symptoms appeared (11).

Various interventions have been proposed to delay the decline of cognitive ability in MCI patients, including pharmacological, non-pharmacological, and multi-component interventions. Since each drug has potential side effects, multiple medications may also lead to increased adverse reactions due to drug interaction. Therefore, non-pharmacological methods are now recommended for the treatment of MCI (6, 12). Existing studies have shown a link between physical exercise and improving cognitive and physical performance levels in the elderly with MCI (13–16).

Exergames is an active game that operates through physical activity in a closed, safe, interactive environment. In the two-dimensional or three-dimensional game environment, the player selects the virtual task, first composed of head-mounted goggles, computers, seats, and videos, and then adds handheld remote controls, infrared cameras, and balance boards to ensure that the player can receive game visual feedback in time. In addition, exergames also use a variety of manipulators (such as bicycles, badminton rackets, etc.) that are very similar to real-life objects, which not only have the advantages of physical exercise but also have the characteristics of interaction and are highly flexible tools. Researchers have proposed the MCI-Game Therapy Experience (MCI-GaTE) framework, which can be used to develop serious games as effective cognitive and physical rehabilitation tools. This framework consists of four components: the abilities of MCI players, core game elements that support gaming and entertainment activities, therapeutic elements that support cognitive and physical rehabilitation through tasks and scenarios, and motivational elements that enhance players’ attitudes toward serious tasks. As a novel cognitive rehabilitation training method, exergames can add the fun that traditional rehabilitation training lacks, potentially increasing the long-term motivation of MCI patients and thus promoting active participation. Furthermore, according to the MCI-GaTE framework, personalized treatment plans can be provided for different MCI patients, which may improve the effectiveness of traditional rehabilitation (17). Many recent studies have used video games as an intervention method; resulting in good clinical efficacy (18–21). Research indicates that exergames can improve cognitive health through several mechanisms. Firstly, mild cognitive impairment is accompanied by a loss of neuroplasticity. As a form of exercise game, exergames involve regular, sustained physical activity (averaging 2–3 h per week over a period of 4–6 months), which can enhance the neuroplasticity of certain brain structures. This increased neuroplasticity can help maintain or even improve cognitive abilities as individuals age (22). Additionally, during physical exercise, cardiac output increases, leading to enhanced blood circulation throughout the body, including the brain, to meet the heightened metabolic demands (23). This augmented cerebral blood flow is correlated with increased neural activity. Recent research demonstrates that elderly individuals who engaged in a structured exercise regimen for 1 year exhibited a significant increase in cerebral blood flow compared to baseline measurements taken a year prior (24). Sun et al. (25) demonstrated that a 24-week Ba Duan Jin exercise program based on exergames effectively improved the quality of life, physical function, and cognitive abilities in elderly individuals with MCI. Thapa et al. utilized a Virtual Reality-Based Intervention Program, conducting four series of cognitive training over 8 weeks for elderly individuals with MCI. Their results indicated positive effects on cognitive functions, with significant improvements in patients’ executive functions (26). Currently, the design of exergame interventions varies across different studies (including the duration, length, and outcomes of the interventions). It is necessary to identify the commonalities and differences among various approaches to filter out universal characteristics. This will provide guidelines for the development of future exergame intervention programs.

This systematic review and meta-analysis aim to describe and determine the effects of video games on different cognitive tasks and explore the impact of video game intervention in the multi-task. Multi-task and single-task modes on the elderly with MCI.

## 2 Methods

### 2.1 Literature search

This article followed the Preferred Reporting Items for (PRISMA) guideline (27). A comprehensive search of the study used PubMed, Web of Science, EBSCO, Elsevier, ProQuest, CNKI, Wanfang and VIP database from inception to April 1, 2024. Two researchers identified keywords and searched them according to the PICOS framework (34). The three main keywords were “mild cognitive impairment,” “cognitive function,” and “exergames.” The search strategy of PubMed is presented in Table 1.

**Table 1:** A database search of PubMed.

#	Searches
1	(((Cognitive Dysfunction (Title/Abstract)) OR (Cognitive Impairments (Title/Abstract))) OR (Mild Cognitive Impairment*(Title/Abstract))) OR (MCI(Title/Abstract))
2	(((“cognitive*”(Title/Abstract)) OR (“cognitive ability “(Title/Abstract))) OR (“cognitive function”(Title/Abstract))) OR (“cognitive decline”(Title/Abstract))
3	(((Exergaming*(Title/Abstract)) OR (Virtual Reality*(Title/Abstract))) OR (Active-Video Gaming (Title/Abstract))) OR (Exergames (Title/Abstract))
4	1 and 2
5	3 and 4
6	Limit 5 to (English language and humans)

### 2.2 Inclusion and exclusion criteria

The literature was included based on the PICOS framework of evidence-based medicine. Five factors were considered: subjects, interventions, control groups, research results, and design. First, two researchers read the title and abstract of the literature, excluding research unrelated to the topic. After removing duplicate documents, the full text was read and the articles were retrieved according to the inclusion criteria: (i) Participants were people aged 60 years or older diagnosed with mild cognitive impairment, regardless of gender; (ii) The experimental design adopted randomized control trials (RCTs); (iii) The intervention segments were exergames with physical activity or exergames as the intervention variable; and (iv) Outcome measures could be any standardized neuropsychological instrument to assess cognitive function, with statistical data including sample size, mean, and standard deviation. Exclusion criteria were as follows: (i) Studies involved the elderly with other psychiatric disorders; (ii) Outcomes were not related to cognitive performance (e.g., overall cognitive function, executive function, memory, etc.); and (iii)The experimental data were incomplete.

### 2.3 Study selection and data extraction

According to the PRISMA statement, two researchers performed literature screening and data extraction using an independent double-blind method based on the proposed literature inclusion and exclusion criteria. First, the study that did not meet the research topic was excluded by reading the title and abstract of the article. After the preliminary screening was completed, the literature that was finally included in the study was determined by reading the full text of the article. If disagreement were encountered, it would be resolved by negotiation; if it could not be negotiated, it would be decided by a third researcher. Two researchers independently extracted and recorded data in a pre-designed Study Data Feature Table. The extraction content mainly included the author’s name, the year of publication, the characteristics of the subjects (such as gender and education level), diagnostic criteria, the number of participants, intervention content, intervention platform, intervention cycle, and outcome measures. Mean values, standard deviation (S.D.), and the number of participants in each group before and after the intervention were extracted from the included articles.

### 2.4 Quality assessment

Two researchers independently completed the quality assessment of each selected literature. Two researchers used the Cochrane Risk of Bias version 2 (RoB2) tools (35) to independently determine the level of risk of each study. The scoring criteria mainly included the following seven areas: random sequence generation (selective bias), allocation concealment (particular bias), blinding of subjects and researchers (implementation bias), blinding of outcome assessment (measurement bias), incomplete outcome data (follow-up bias), selective reporting (reporting bias), and other bias. A third investigator decided that disagreements could not be resolved to reach a consensus and ultimately determine the quality of the literature. Each project was evaluated with “low risk,” “high risk,” and “unclear.”

### 2.5 Statistical analysis

RevMan 5.4 software recommended by Cochrane Collaboration network and Stata version 12.0 (Stata Corp) were used for statistical analysis. Since the included studies had measured scores from baseline to post-intervention, which were continuous variables, the Mean difference (MD) and 95% confidence intervals (CIs) were calculated based on the mean, standard deviation, and sample size of the intervention and control groups of outcome indicators, and the influence of each study on the combined results was determined by weight. If *p* < 0.05, the difference was statistically significant. The *I*^2^ statistic was chosen as the measure of heterogeneity. The *I*^2^ statistic is a commonly used measure that indicates the proportion of variation observed between studies. An *I*^2^ value of 0% indicates no observed heterogeneity. Generally, an *I*^2^ value greater than 56% suggests substantial heterogeneity among the studies, with larger values indicating greater heterogeneity. The selection of the model is based on the *I*^2^ value: a random effects model is chosen when statistical heterogeneity is significant (*I*^2^ > 50%), whereas a fixed effects model is chosen when statistical heterogeneity is not significant (*I*^2^ ≤ 50%). If there is a combination result with large heterogeneity (generally thought to be *I*^2^ > 75%), sensitivity analysis is conducted to find the source of heterogeneity. Publication bias was assessed using funnel plots.

## 3 Results

### 3.1 Search results

**Figure 1:**
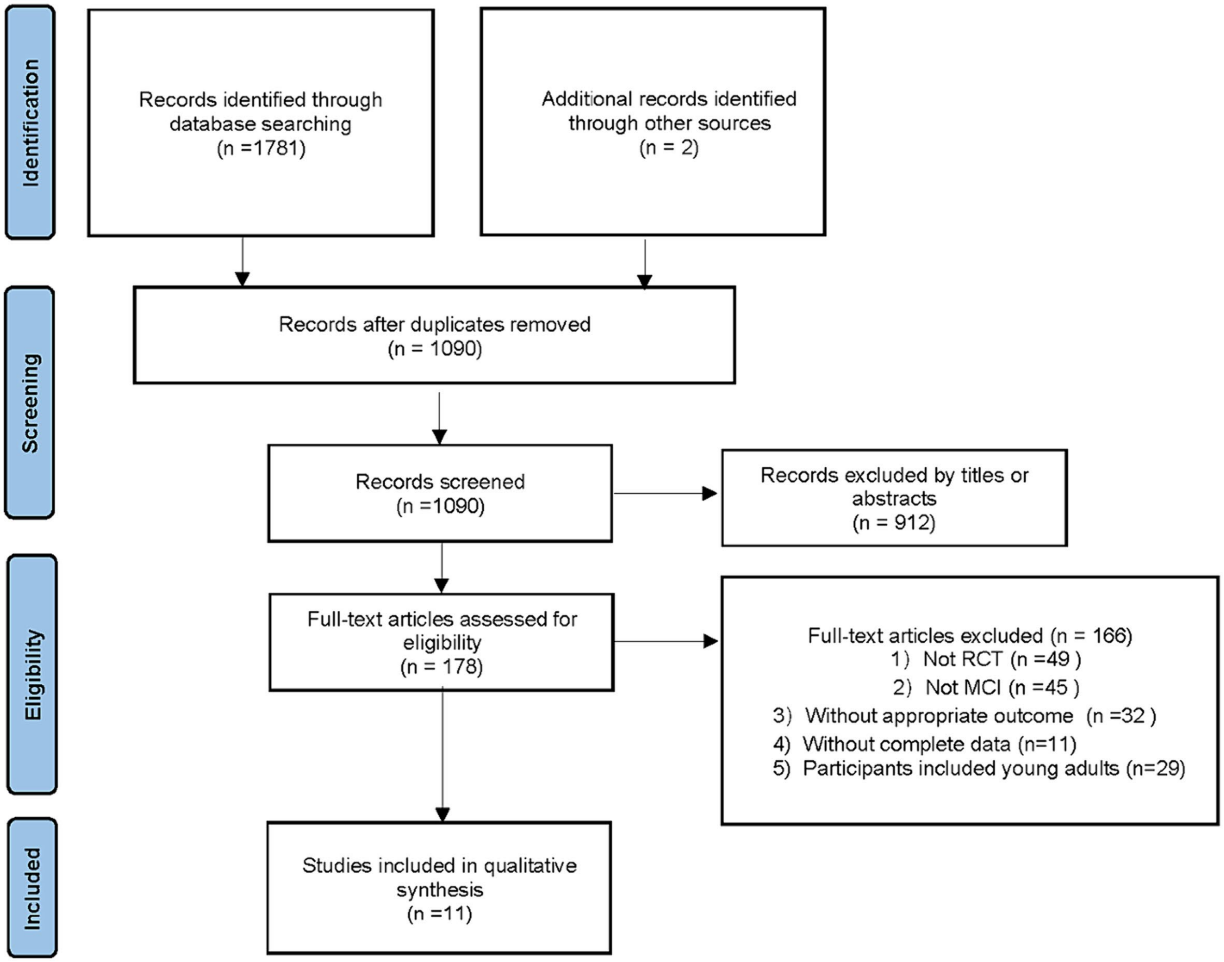
Study flow diagram.

Through a preliminary search, we obtained 1,783 related studies from Chinese and foreign databases and two additional records through other sources (through Google Scholar search). Among them, 695 publications were screened by two researchers independently through reading titles and abstracts and were eliminated due to repeated publication. After reading the titles and abstracts of 1,090 papers, 912 articles that did not meet the inclusion criteria were excluded. The remaining 178 articles were read in full text, and 11 were finally included, involving 526 subjects (Figure 1).

### 3.2 Characteristics of included literature

Among the 11 pieces of RCT literature included, two were Chinese and nine were English. All the literature used V.R. technology to intervene in the experimental group, and different experiments included different numbers of intervention items.

Most studies have conducted some intervention in the control group, including physical activity training in five studies, routine cognitive training in three studies, and usual nursing in one study; In addition, two projects did not intervene in the control group. The duration of exercise intervention for the intervention group ranged from 6 to 24 weeks, and each study had its own intervention time and frequency (Table 2).

**Table 2:** The general characteristics of the studies included.

Included studies	Mean age (Years)	Diagnostic criteria	Education level	Participants (M/W)	Sample size (*N*)	Session Frequency	Intervention	Task content	Platform	Intervention duration (weeks)	Outcome measure
Sun et al., 2021 (27)	65–85	MMSE>24; MoCA<26	IG: PS (9); JHS (9); HSATSS (6); U (5)	57(21/36)	IG = 29; CG = 28	IG:5 min Warm-up+20 min V.R. training +5 min Relaxation training, three times a week	Single-task	VR Baduanjin Exercise	V.R. training system (Nanjing, version 2.0 of MuoXun)	24	MoCA; TMT-A; TMT-B
			CG: PS (7); JHS (10); HSATSS (7); U (4)			CG: Routine rehabilitation					
Choi and Lee, 2018 (19)	76.32 ± 4.17	MoCA<26	NM	60(9/51)	IG = 30; CG = 30	IG: 10 min warm-up	Single-task	Virtual Kayak Paddling Exercise	TheraBand Exercise Station, (Hadamar, Germany)	6	MoCA
						40 min V.R. training+10 min Relaxation training, two times per week					
						CG: Maintain daily activitie					
Delbroek et al., 2017 (28)	87.2 ± 6.1	MoCA<26	NM	20(7/13)	IG = 10; CG = 10	EG: 18 min VR training in week 1	Multi-task	VR training (Downhill ski; Weight-bearing transfer; Avoidance while walking;Memory exercise)	BioRescue (RM Ingenierie, France)	6	MoCA
						gradually increased to 30 min in week 5, twice a week					
						CG: Maintain daily activities					
Tarnanas et al., 2015 (29)	71.35 ± 11.69	MMSE≥25	IG:16.1 ± 6.4	78(50/28)	IG = 40; CG = 38	IG:20 min VR training	Multi-task	VR training (Emergency evacuation)	NM	12	MMSE
			CG:15.6 ± 8.0			CG:No other treatment					
Mrakic-Sposta et al., 2018 (30)	73.3 ± 5.79	MMSE≥25	NM	10(4/6)	IG = 5; CG = 5	IG: V.R. training increased from 40 min in the first 3 weeks to 45 min in the latter, three times a week	Multi-task	VR training (Riding a bike, Crossing roads; Grocery shopping)	PlayStation controller (Sony, Japan)	6	MMSE; TMT-A
						CG: No other treatment					
Tarnanas et al., 2014 (31)	70.7	Clinical evaluation	NM	66(25/41)	IG = 32; CG = 34	IG: 90 min V.R. training, twice a week	Multi-task	VR Museum Cognitive Training (Finding objects; Story recollection; Archeological practice)	Virtual Museum system (Developed by XML and VRML)	20	MMSE; TMT-B; DSF
						CG: conventional cognitive training					
Park et al., 2020 (21)	76.5 ± 7.9	MMSE>16	IG:UN(2);ES(13); MS (2); HS (1)	35(17/18)	IG = 20; CG = 20	IG: 30 min V.R. training, five times a week	Multi-task	VR training (Driving; Door opening; Bathroom time)	The MOTOCOG® system (Cybermedic Inc., Korea)	6	MoCA; TMT-A; TMT-B; DSF
			CG:UN(1);ES(13); MS (2); HS (1)			CG: Conventional cognitive rehabilitation					
Tarnanas et al., 2014 (31)	72.5 ± 5.32	Clinical evaluation	IG.: 9.3 ± 4.0	68(16/52)	IG = 34; CG = 34	IG: 60 min V.R. training+30 min Eye massage, three times a week	Multi-task	VR training (Juice making; Crow Shooting; finding the fireworks number; memory object)	NM	8	MMSE
			CG:8.4 ± 3.5			CG: Physical exercise course					
Liao et al., 2019 (30)	74.3 ± 6.0	MMSE>24; MoCA<26	IG: 9.3 ± 3.8	34(23/11)	IG = 18; CG = 16	IG: 3 times a week	Multi-task	VR training (Take the MRT; Kitchen chef; Convenience store clerk; Tai Chi; Football)	Kinect	12	TMT-A; TMT-B
			CG:9.9 ± 2.1			CG: Conventional physical and cognitive training					
Amjad et al., 2019 (32)	NM	Clinical evaluation	NM	38(NM/NM)	IG = 18; CG = 20	IG: 5 min Warm-up+25 min VR training,+10 min Relaxation training, 2 times per week	Multi-task	VR training (Logic;Physical; Memory; Reflexes and Math)	Kinect for Xbox 360	6	MMSE; MoCA
						CG: Physical exercise course					
Hu et al., 2018 (26)	74.1 ± 5.8	19≦MoCA≦25	NM	60(47/13)	IG = 30; CG = 30	IG.: V.R. training was gradually extended from 5 min at the beginning to 15 min, once a day	Multi-task	VR training (Domestic activities; Kitchen cooking; Cycling)	BioMaster	12	MoCA
						CG: Routine rehabilitation					

M, Man; W, Woman; IG., Intervention group; CG, Control group; NM., Not mentioned; PS, Primary school; JHS, Junior HS; HSATSS, HS and technical secondary school; U, University; UN, Uneducated; ES, Elementary school; MS, Middle school; HS, High school; XML, eXtended Markup Language; VRML, Virtual Reality Markup Language; MMSE, the Mini-Mental State Examination; MoCA, the Montreal Cognitive Assessment; TMT, the Trail Making Tests; DSF, Digit Span Forward.

### 3.3 Risk of bias assessment

The risk of bias graphs for all included studies shows the judgment of bias risk items in the included studies according to the Cochrane Risk of Bias version 2 (RoB2) tools. The quality of the 11 studies included at this stage was relatively high, among which seven experiments reported the generation of random sequence, and four studies reported the method of assigning hiding. Still, the intervention received by the subjects was exercise intervention; the experimenters could not be blinded, so there was a high risk of implementation bias in this study. All other biases in 11 studies were judged and classified as potential low-risk bias.

### 3.4 Results of meta-analysis

**Figure 2:**
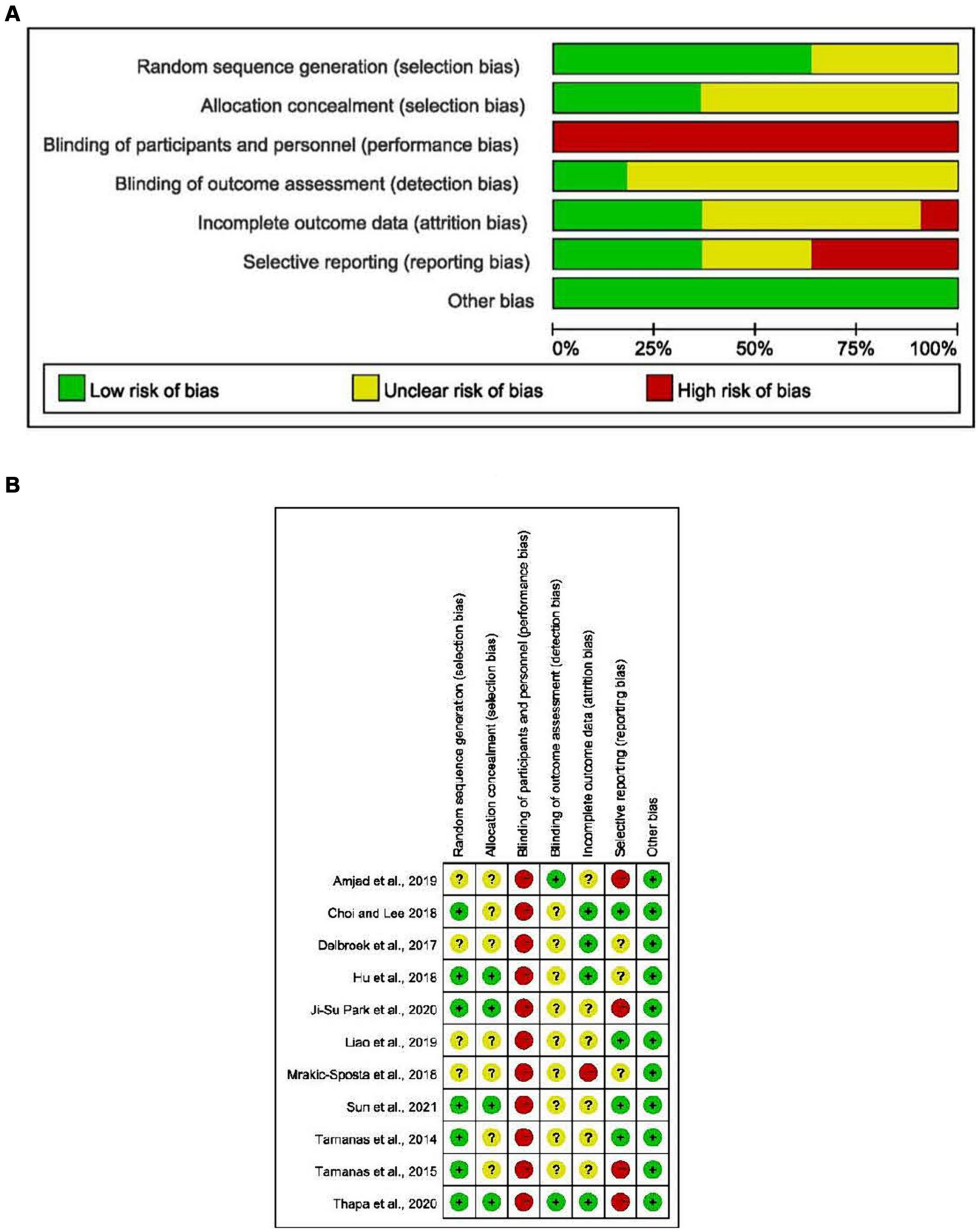
**(A)** Risk of bias summary; **(B)** risk of bias assessments.

This review synthesized data from studies using the same outcome measures for a meta-analysis of 11 studies. Outcomes of cognitive ability assessment for meta-analysis included: the Mini-Mental State Examination (MMSE), the Montreal Cognitive Assessment (MoCA); the Trail Making Tests-A & B (TMT-A & B); Digit Span Forward (DSF). The collation of literature information found that the intervention methods of the experimental group were mainly divided into single-task and multi-task (Figure 2).

The MMSE scale was measured in four studies, including 120 subjects in the intervention group and 104 subjects in the control group. The results showed that the MMSE score of the intervention group was significantly higher than that of the control group (MD = 2.05, 95% CI = 0.59–3.51, *p* = 0.06, *I^2^* = 78%).

In the overall analysis, the MoCA scale was measured in seven studies, 135 subjects were in the intervention group and 1,312 subjects were in the control group. The results showed that the combined results between the control and intervention groups were not statistically significant (MD 1.87, 95% CI − 1.04 to 4.78, *p* = 0.21, *I^2^* = 97%).

Trail Making Tests-A was measured in three studies, including 65 subjects in the intervention group and 61 subjects in the control group. The results showed that compared with the intervention group, the TMT-A score of the control group was higher (MD = −3.89, 95% CI = −6.45 to −1.33, *p* = 0.003, *I^2^* = 0%). There was no heterogeneity among TMT-A studies.

Trail Making Tests-B measurement was performed in four studies, including 97 subjects in the intervention group and 95 subjects in the control group. The results showed that there was no significant difference in TMT-B scores between the intervention group and the control group (MD = −34.58, 95% CI = −84.83 to 15.66, *p* = 0.18, *I^2^* = 96%).

**Figure 3:**
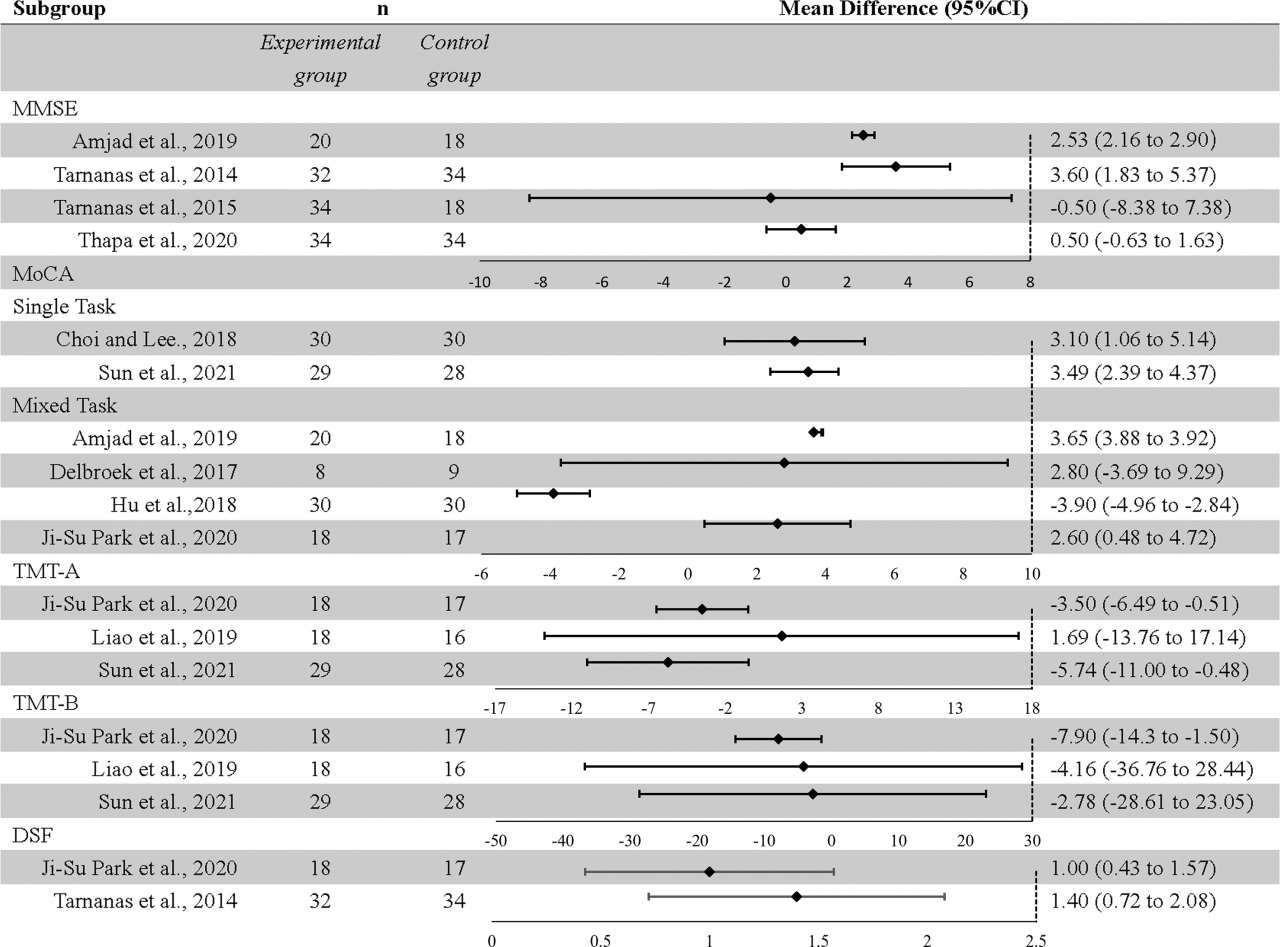
Effect of exergames on scale outcomes in overall analysis: forest plot.

Two studies provided DSF measurement results, including 50 subjects were in the intervention group, and 51 subjects were in the control group. The results showed that the DSF score of the intervention group was significantly higher than that of the control group (MD = 1.16, 95% CI 0.73–1.60, *p* < 0.00001, *I^2^* = 0%) (Figure 3).

### 3.5 Subgroup analysis

When the intervention group was intervened, the effect of the different number of intervention tasks on overall cognition was reflected in the meta-merged results of MoCA scale scores. Two studies reported single-task intervention (S.T.), including virtual kayaks and virtual Baduanjin. Four studies reported multi-task intervention (M.T.), especially the effect of physical activity combined with cognitive training. Compared with the control group, the overall cognitive level of the intervention group receiving S.T. was significantly higher. (MD 3.40, 95% CI 2.43–4.37, *p* < 0.00001, *I^2^* = 0%). However, the effect of M.T. on overall cognition was not statistically significant (MD 1.15, 95% CI −3.70 to 6.00, *p* = 0.64, *I^2^* = 98%), and there was high heterogeneity among studies.

### 3.6 Sensitivity analysis

**Figure 4:**
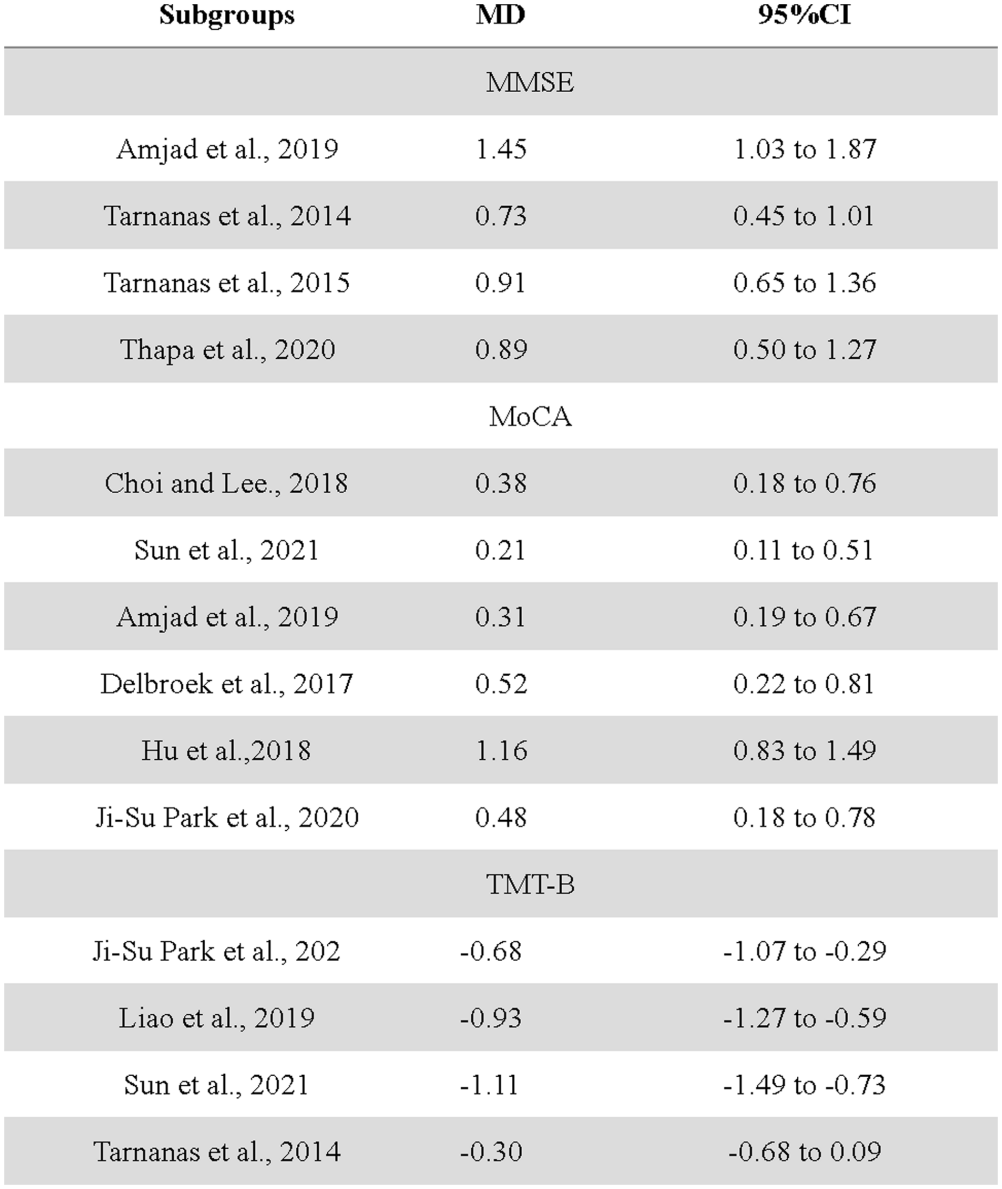
The impact of each study on scale outcome.

**Figure 5:**
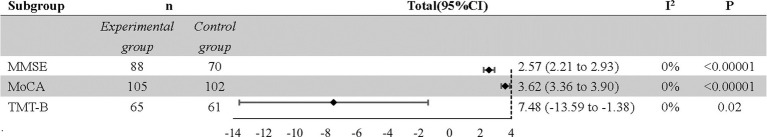
Forest plots of scale outcome in overall analysis after culling.

Due to the high heterogeneity observed in the outcomes of the MoCA scale, MMSE scale, and TMT-B test, this study employed a leave-one-out sensitivity analysis. This approach was used to examine the influence of each individual study on the overall effect estimate, to understand the heterogeneity among study results, and to identify potential sources of this heterogeneity. The MMSE scale showed high heterogeneity between studies (*I*^2^ = 78%), with four studies including MMSE being excluded one by one, and Thapa 2020 was removed for meta-analysis. The sensitivity analysis results showed that there was no heterogeneity among the studies, and the combined results were statistically significant (95% CI 2.21–2.93, *I^2^* = 0%). In the measurement results of the MoCA scale, there was high heterogeneity among the studies (*I^2^* = 97%). Six studies containing MoCA were eliminated one by one, Hu2018 was eliminated, and meta-analysis was combined. The results showed that there was no heterogeneity among the studies, and the combined results were statistically significant (95% CI 3.36–3.87, *I^2^* = 0%). In the measurement results of TMT-B, there was high heterogeneity among the studies (*I^2^* = 96%). Six studies including TMT-B were eliminated one by one, Tarnanas was eliminated, and meta-analysis was combined. The results showed that there was homogeneity among the studies, and the combined results were statistically significant (95% CI −13.59 to −1.38, *I^2^* = 0%). Therefore, the meta-analysis of this study is robust (Figures 4, 5).

### 3.7 Publication bias

After sensitivity analysis, visual examination of five different funnel plots showed that most of the studies focused on the top, and the symmetry of the left and right tops was acceptable, indicating that there was little possibility of publication bias in the cognitive function of the elderly.

## 4 Discussion

This systematic review and meta-analysis synthesize the effects of exergames on the cognitive ability of MCI patients in 11 studies. Results from various cognitive tests (MMSE, MoCA, TMT-A, TMT-B, and DSF) were extracted and analyzed to evaluate the changes in cognitive ability after exergames-based interventions (EBI). The results showed that exergames can effectively improve the cognitive function of MCI older adults.

The overall analysis of the scores of each scale showed that sports games had a significant effect on the improvement of cognitive function. Specifically, in the non-heterogeneous intervention group, exergames positively affected cognitive ability by reducing TMT-A scores and increasing DSF scores, including attention, cognitive speed, and working memory. Due to the differences in the exergames programs in the included studies, this study divides the intervention methods into single and multi-tasks. The results of the subgroup analysis showed that compared with the control group, single-task intervention could positively affect the overall cognitive ability by improving MoCA scores. Our results were consistent with previous EBI studies (12, 18). The overall comprehensive results obtained in this study are optimistic, but it is important to explain that in some other cognitive tests, such as the Montreal Cognitive Assessment (MoCA), Trail Making Test B (TMT-B), and Digit Span Forward (DSF), no significant impact of the action video game intervention was observed. Specifically, both the MoCA and TMT-B demonstrated favorable overall effects after eliminating heterogeneous sources. The comprehensive results of the Digit Span Forward (DSF) test indicated that there was no significant differential positive effect on cognitive function between the motion group and the control group. This result aligns with previous research. Existing studies suggest that, compared to the Digit Span Forward (DSF) test, the Digit Span Backward (DSB) test, using the Corsi Span task (CS) to assess improvement in short-term memory and attention in individuals with mild cognitive impairment, yields more significant results (36). This could provide some reference for the selection of outcome indicators in future intervention trials.

Central nervous system function declines irreversibly with age, and most studies suggested that physical activity is associated with improved neuropsychological function. Some studies have found that the change in carotid artery elasticity and the imbalance of vasoconstriction and diastolic function will aggravate the degree of cognitive impairment. Physical activity, especially aerobic exercise, can increase cerebral blood flow, glucose utilization, oxygen extraction and activation of capillary density and other structural changes of growth factors to improve cardiovascular system function. Available data support that the frontal effect of exergames therapy may be related to the reactivation of several brain neurotransmitters such as cholinergic and dopaminergic. Promoting the process of neural plasticity may reverse the degenerative changes of nerves and have a beneficial effect on the cognitive function of the elderly (37–41).

Due to the high heterogeneity in the comprehensive analysis of forest plot results using MMSE, MoCA, and TMT-B as outcome indicators, sensitivity analysis by excluding one study found that heterogeneity in the outcomes of the EBI in the MMSE, MoCA, and TMT-B scales was due to one study included in the analysis. The comprehensive analysis conducted after excluding sources of heterogeneity yields more reliable results. Specifically, the intervention period of Thapa et al. (26) in the MMSE scale analysis was relatively short (8 weeks), but the frequency was high (100 min per time, three times a week). Compared with the interventions in the other three studies, there are obvious methodological differences, which could be speculated as the influencing factors of heterogeneity. In the multi-task intervention subgroup of the MoCA scale analysis, Hu et al. (42) (31) (29)selected elderly patients with COPD and MCI. Compared with several other studies, subjects’ inclusion and exclusion criteria in clinical trials were significantly different. In addition, the intervention platform used in the trial was the BioMaster platform, which was different from other studies. Moreover, the intervention group was accompanied by conventional drug treatment, and the type of drug was not explained in detail in the article. It was not excluded that drugs for MCI treatment would inevitably affect the results of EBI experiments. Tarnanas et al. (31) in the TMT-B scale analysis, the intervention group also received multiple cognitive training combined with virtual reality training. The virtual reality intervention was conducted one-to-one, while other research intervention groups only conducted video game intervention. The difference in intervention methods was the specific cause of heterogeneity. In the non-heterogeneous intervention group, the results showed that the intervention of exergames positively impacted executive ability in the sub-cognitive domains. The previous meta-analysis suggested that positive exergames were beneficial to the improvement of executive function and visual–spatial skills. The results were consistent (19). Moreover, since the studies included in this metaanalysis are all RCTs, the random assignment of participants to different groups effectively controls for confounding factors, thereby reducing bias. During the implementation of the trials, blinding was not applied to the participants because the control group did not undergo any treatment changes. As a result, it is possible that participants in the intervention group were aware of their group assignment, which might have led to more active participation in the treatment or higher expectations of its efficacy, potentially influencing their responses and the reported outcomes.

The most interesting finding in the subgroup analysis was that in the non-heterogeneous intervention group, the S.T. intervention significantly improved overall cognitive ability. Specific cognitive training and a vivid environment have also been shown to enhance cognitive performance and improve brain health in the elderly (43, 44). The included S.T. EBI study shares commonalities in task design and implementation. Choi et al.’s exercise regimen was conducted as a group exercise within a well-controlled VR environment, with a coach providing guidance throughout the intervention. Similarly, Sun et al. selected Ba Duan Jin as the intervention exercise. Before the official intervention, a coach conducted group training for 1 week to ensure proper form. During the intervention, a virtual coach (via a programmed system and cameras) continuously guided participants’ movements (25, 45). The advantages of group practice include adherence to a predetermined schedule at specific times and locations, which may positively influence participants’ compliance. Additionally, the supervision by a coach may enhance participants’ enjoyment of the exercise and their self-efficacy. Furthermore, both kayaking exercises and Ba Duan Jin require participants to maintain focus and concentration during the activities, which, compared to simple repetitive movements, are more likely to stimulate cognitive processes. These elements may be effective in improving cognitive functions in MCI patients.

It is well known that drug therapy and exercise therapy have always been the main intervention targets for cognitive diseases. There is no clear evidence to support that the drug can be discontinued during the treatment when a certain course of treatment is reached or when disease progression changes. Because long-term drug treatment will produce high treatment costs and unavoidable side effects, and there is a certain clinical delay in drug treatment, it is necessary to find other effective treatment methods (40). McEwen et al.’s studies on physical exercise and cognitive disorders have suggested that, as long as the intensity and duration of exercise intervention are sufficient, there are usually positive exercise results. One aspect of long-term physical activity interventions associated with improved cognitive function is adherence to the intervention (46–48). In this sense, stroke patients activate ipsilateral brain regions related to controlling affected hands by reshaping the sensory-motor cortex after video game intervention. As an auxiliary means of conventional treatment, exergames can effectively improve the functional outcome of stroke. Video game-based exercise training improves the ability of Parkinson’s patient’s brains to perceive, process, and integrate information and helps patients better maintain balance and posture control.

Exergames systems can form personalized patient treatments through special software programs 、input and output devices. According to the needs of rehabilitation and patients, it provides rehabilitation programs with different difficulties and forms. Many commercial VR systems are interesting and can be used as a daily pastime. It can attract and stimulate the enthusiasm of patients to participate (49, 50). Many studies have also reported high compliance with video game interventions (51). In the future, practitioners can gradually incorporate exergames as a treatment modality for cognitive function in patients with MCI. It is necessary to integrate exergames into the treatment plans for MCI. At this stage, a personalized exergame therapy plan can be developed through three phases: assessing needs, devising a plan, and continuous monitoring (52). This can complement existing pharmacological treatments or other cognitive therapies. However, it is important to consider that the purchase and maintenance costs of exergame equipment can be high. More affordable options, such as the Nintendo Wii, could be utilized (53). Given that exergames are relatively new, it is crucial to ensure that patients can conveniently access and use the equipment. Additionally, before initiating interventions, both patients and healthcare providers may need to undergo appropriate training to correctly use the equipment and understand the game rules.

Nowadays, exergames constitute a new frontier of rehabilitation and are considered to be an innovative and economical physical activity that can promote the cognitive level of the elderly (18, 50). However, it still faces the obstacle of becoming a widely used tool in psychiatric practice. First of all, due to the small sample size and the small number of studies, the available evidence suggests that exergames emphasize customized training courses for patients to achieve the best results, which limits the consensus on the effectiveness of video game intervention. Besides, it is necessary to consciously overcome technical defects such as eye dryness, motion sickness, and user obstacles such as addiction (21, 49). It should be emphasized that exergames as exercise training, MCI patients must exercise scientifically and reasonably. Physical therapists are required to control the intensity and frequency of interventions, and it is necessary to be accompanied by professionals.

Currently, most studies on exergame interventions for MCI patients focus on popular exercise games such as Wii Fit or Dance Revolution. Future research could explore a wider variety of exercise games, including augmented reality (AR) games or those using new interactive devices like Kinect. Additionally, researchers could investigate games specifically designed for cognitive function training, such as memory games and attention training games, to create more targeted interventions. Most existing studies are short-term; thus, future research should examine the long-term effects of exercise games on cognitive function. Given the commercial nature of many exercise game technologies, future studies should place greater emphasis on managing conflicts of interest. This includes transparently disclosing all potential conflicts and ensuring the independence of the research.

## 5 Limitations

This systematic review and meta-analysis followed the PRISMA statement list, but there were still some limitations to note. First of all, the scope of literature retrieval only included Chinese and English literature, and there was a possibility of missing minority languages and gray literature. Secondly, the analysis results of some outcome indicators were highly heterogeneous, and specific speculation and explanation were needed. In addition, the intervention cycle, intensity and platform of EBI used in the included studies were different. Therefore, there was a certain heterogeneity in the intervention, and it was not easy to draw general recommendations on using exergames rehabilitation. The same EBI system should be used as an observation index in future research. Moreover, due to the lack of available follow-up data in the included studies, this research cannot track the long-term sustainability of cognitive improvement post-intervention. Future studies should incorporate long-term evaluations of cognitive enhancement to address this limitation.

## 6 Conclusion

Exergames have the potential to significantly enhance cognitive function in elderly individuals with MCI. However, the findings of our study remain inconclusive due to three primary factors. Firstly, this meta-analysis exclusively RCTs. Secondly, the majority of exergames are unable to fully replace existing MCI treatment modalities. Thirdly, current intervention studies have predominantly examined the short-term effects of exergames on elderly individuals with MCI. It is imperative for game developers to consider the limited sample sizes utilized in current meta-analyses and to recognize the necessity for developing more effective exergames, particularly those based on VR or other mobile devices, for MCI intervention. At present, exergames serve as a supplementary tool to existing MCI treatments. To enhance the generalizability and effectiveness of exergame interventions, future research should undertake comprehensive comparisons of (1) EBI across different intervention durations, (2) EBI versus traditional MCI interventions, (3) different types of EBI, such as cognitive training games and exercise games, and (4)Non-Randomized Studies.

## Data availability statement

The original contributions presented in the study are included in the article/Supplementary material; further inquiries can be directed to the corresponding author.

## Author contributions

XC: Writing – original draft. LX: Writing – review & editing. HZ: Writing – review & editing. TS: Writing – review & editing, Writing – original draft. JY: Writing – review & editing. XJ: Writing – review & editing. XH: Supervision, Writing – review & editing. RS: Supervision, Writing – review & editing. JP: Writing – review & editing.

## Glossary

MCI: Mild cognitive impairment
CNKI: China National Knowledge Infrastructure
MD: Mean difference
95% CI: 95% confidence interval
EBI: Exergames-based interventions
RCTs: Randomized control trials
S.D.: Standard deviation
S.T.: Single-task intervention
DSB: The Digit Span Backward
CS: The Corsi Span task
MMSE: Mini-Mental State Examination
MoCA: The Montreal Cognitive Assessment
TMT-A & B: The Trail Making Tests-A & B
DSF: Digit Span Forward
M: Man
W: Woman
IG.: Intervention group
CG: Control group
NM.: Not mentioned
PS: Primary school
MS: Middle school
HS: High school
JHS: Junior HS
HSATSS: HS and technical secondary school
U: University
UN: Uneducated
ES: Elementary school
XML: eXtended Markup Language
VRML: Virtual Reality Markup Language
