# Supplementary material for: The effects of exergames for cognitive function in older adults with mild cognitive impairment: a systematic review and metaanalysis
Source: Front Neurol. 2024 Jul 16;15:1424390. doi: 10.3389/fneur.2024.1424390 (PMC11286570; doi:10.3389/fneur.2024.1424390)
Supplement: Supplementary file 1 [file Data_Sheet_1.PDF]

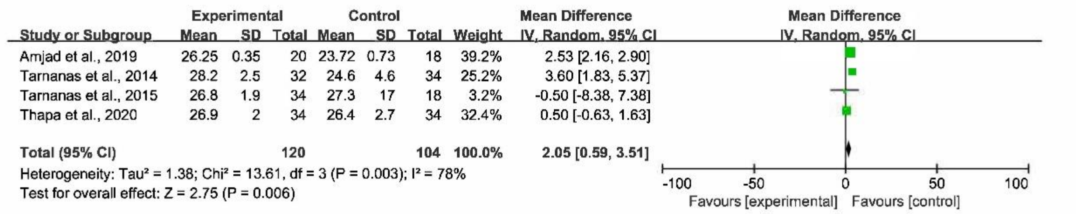

Supplementary figure -1.1: Effect of exergames on MMSE scale outcomes in overall analysis: forest plot

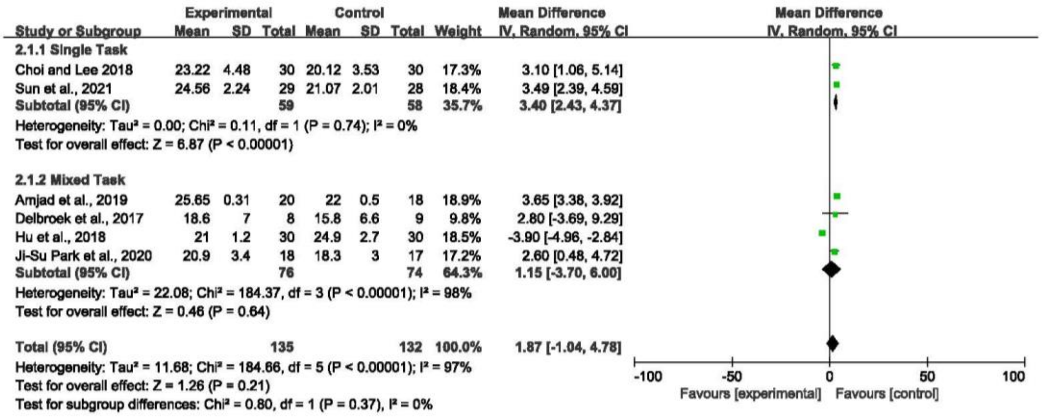

Supplementary figure -1.2: Effect of exergames on MoCA scale outcomes in overall analysis: forest plot

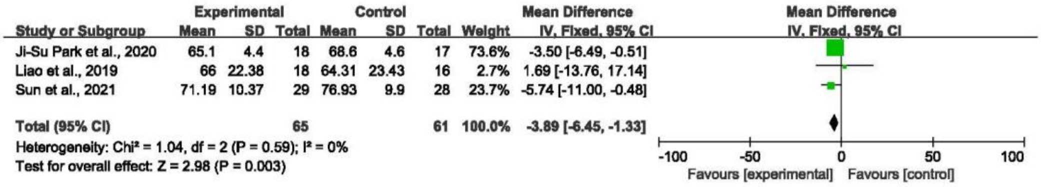

Supplementary figure -1.3: Effect of exergames on TMT-A scale outcomes in overall analysis: forest plot

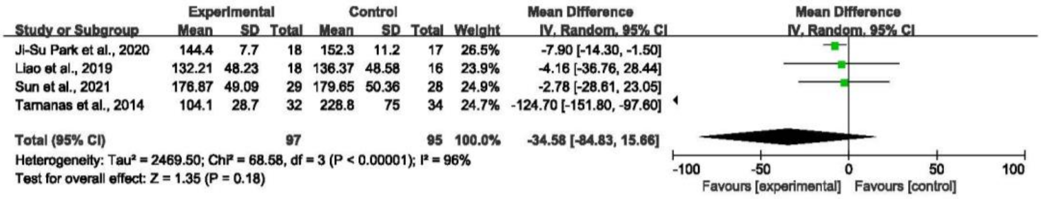

Supplementary figure -1.4: Effect of exergames on TMT-B scale outcomes in overall analysis: forest plot

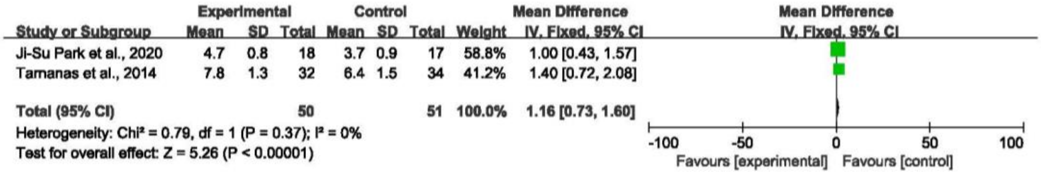

Supplementary figure -1.5: Effect of exergames on DSF scale outcomes in overall analysis: forest plot

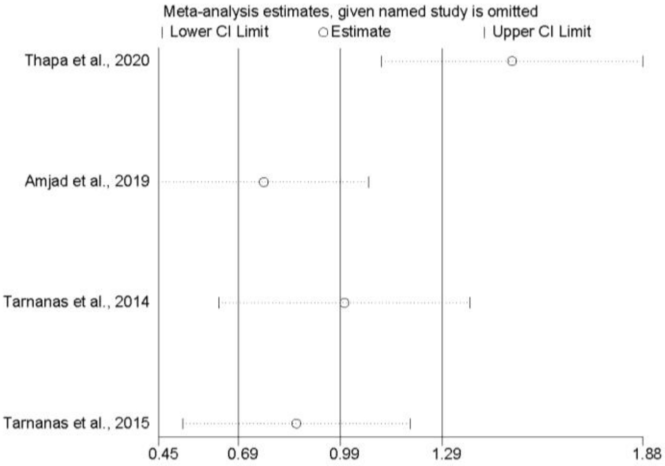

Supplementary figure -2.1: Sensitivity analysis of the impact of each study on MMSE scale outcomes

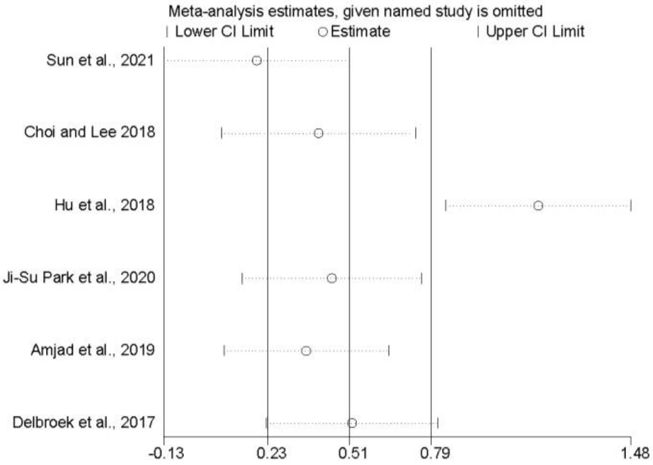

Supplementary figure -2.2: Sensitivity analysis of the impact of each study on MoCA scale outcomes

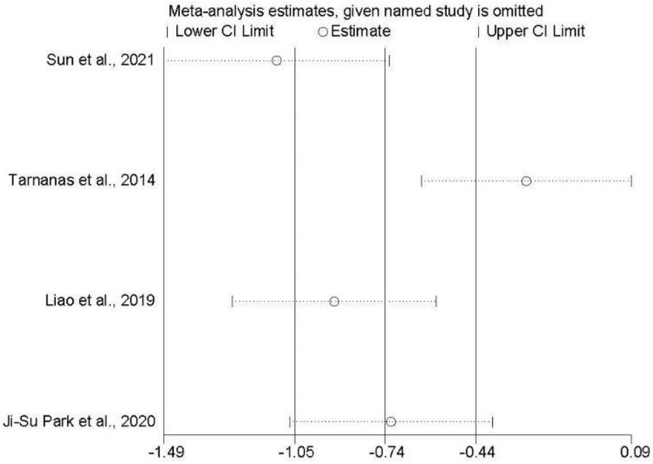

Supplementary figure -2.3: Sensitivity analysis of the impact of each study on TMT-B scale outcomes

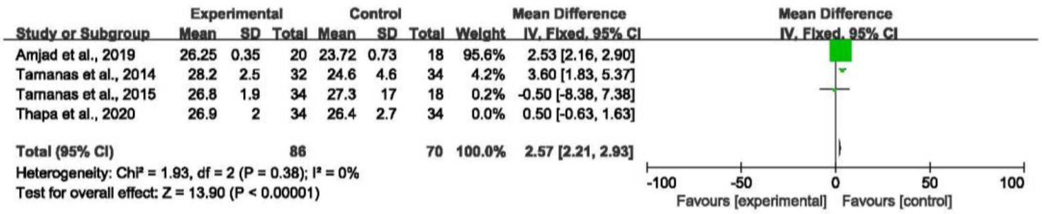

Supplementary figure -3.1: Forest plots of MMSE scale outcomes in overall analysis after culling

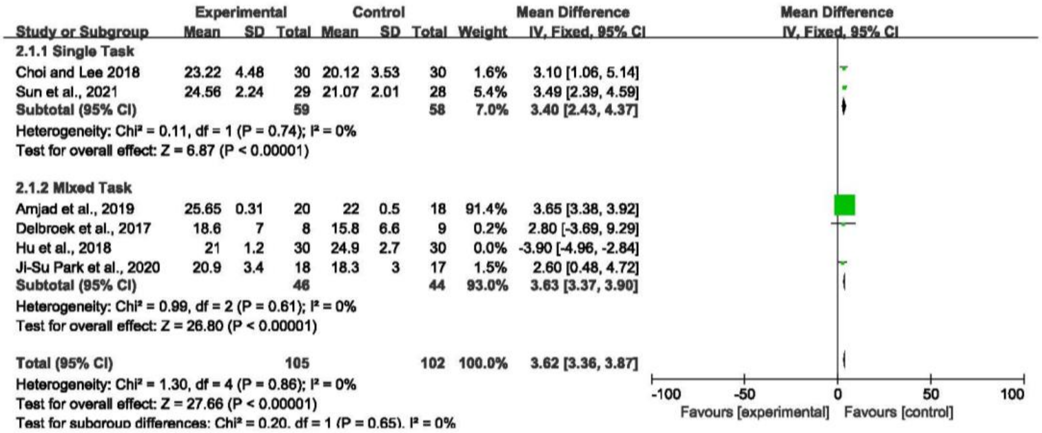

Supplementary figure -3.2: Forest plots of MoCA scale outcomes in overall analysis after culling

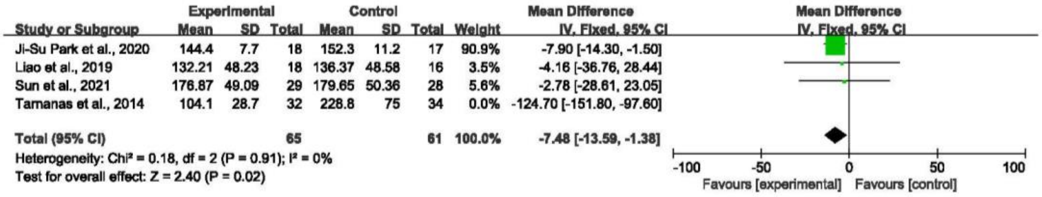

Supplementary figure -3.3: Forest plots of TMT-B scale outcomes in overall analysis after culling

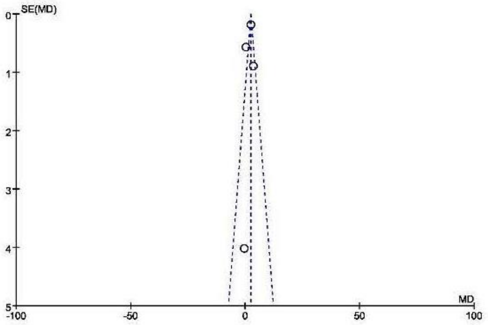

A. MMSE

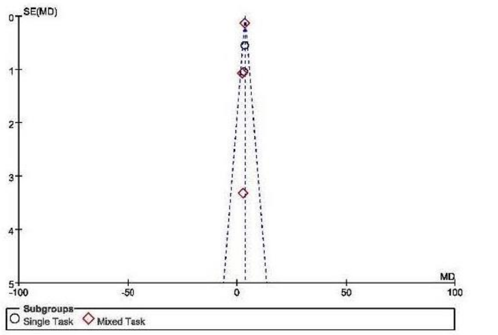

B. MoCA

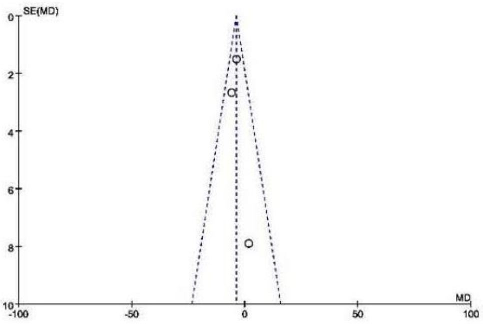

C. TMT-A

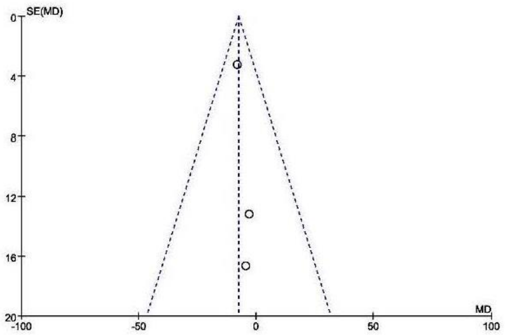

D. TMT-B

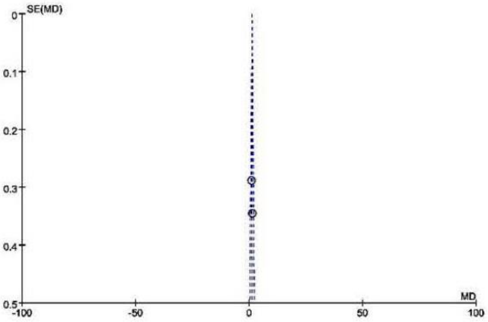

E. DSF

Supplementary figure -4: Funnel plot assessing the publication bias
